# Impact of Losartan on Portal Hypertension and Liver Cirrhosis: A Systematic Review

**DOI:** 10.7759/cureus.83309

**Published:** 2025-05-01

**Authors:** Deepti Avasthi, Nicholas Zerilli, Fahad Shaikh, Taimoor Jamil, Daniyal Ishtiaq, Salil Avasthi

**Affiliations:** 1 Internal Medicine, Mercy Health - St. Vincent's Medical Center, Toledo, USA; 2 Pulmonary and Critical Care Medicine, Mercy Health - St. Vincent's Medical Center, Toledo, USA

**Keywords:** hepatic venous pressure, hepatic venous pressure gradient, liver cirrhosis, liver fibrosis, losartan, portal hypertension, wedged hepatic venous pressure

## Abstract

Portal hypertension, a complication of chronic liver disease, results from an elevated pressure gradient between the portal vein and the inferior vena cava. While non-selective beta-blockers are established for reducing portal pressure, the efficacy of losartan, an angiotensin II receptor blocker, remains debated. This study evaluated losartan's impact on portal pressure and liver fibrosis in patients with cirrhosis and portal hypertension.

The goal of this meta-analysis was to appraise evidence on the role of losartan in reducing portal pressure and associated clinical outcomes in cirrhotic patients with portal hypertension.

A comprehensive literature search was conducted in PubMed, Cochrane Library, Medline, and Web of Science. All the research and literature review were conducted from August 20th, 2024, through August 31st, 2024 (within one month of the paper's submission). The Risk of Bias Visualization Tool (Robvis 2.0) and Risk Of Bias In Non-randomized Studies - of Interventions (ROBINS-I) were used to assess study quality. Data were extracted and analyzed using Microsoft Excel.

Among 426 potential studies, 12 met the inclusion criteria. Both losartan and propranolol reduced hepatic venous pressure gradient (HVPG), with some studies suggesting a more pronounced effect of losartan. A meta-analysis found no significant difference in HVPG reduction (p = 0.22), but losartan significantly reduced wedged hepatic venous pressure (WHVP) compared to propranolol (p = 0.03). Losartan also affected mean arterial pressure, renal function, and hepatic fibrosis.

Losartan shows potential in treating portal hypertension by reducing portal pressure and fibrosis. It may be particularly beneficial in the treatment of liver cirrhosis by addressing both hemodynamic and structural components and improving sodium handling in complex cases.

## Introduction and background

Portal hypertension is a serious complication of chronic liver diseases that can lead to life-threatening conditions like variceal bleeding and ascites. It arises from an increased pressure gradient between the portal vein and the inferior vena cava, often due to elevated intrahepatic vascular resistance and vasodilation of splanchnic blood vessels [1]. This condition can cause liver distortions, secondary fibrosis, and elevated portal blood pressure, particularly in cirrhosis [2]. The pathogenesis of liver cirrhosis is strongly associated with cardiovascular morbidity and shares a common pathologic pathway [3].

Current treatment primarily involves non-selective beta-blockers to reduce portal pressure [4]. Non-selective beta-blockers reduce portal pressures through both beta-1 receptor blockade and beta-2 receptor blockade. Beta-1 blockade is due to decreases in the following: pulse rate, cardiac output, portal venous flow, portal venous pressure, and the gradient between portal venous pressure and free hepatic venous pressure [5]. Beta-2 blockade results in unopposed alpha-1 receptor activation, resulting in splanchnic vasoconstriction [6].

Although non-selective beta-blockers are the first-line treatment, not all patients have optimal hepatic venous pressure gradient (HVPG) response (a decrease of more than 20% of HVPG from baseline or less than 12 mm Hg) to treatment. In a meta-analysis by Turco et al., cirrhotic patients without ascites showed that roughly 50% of patients had optimal HVPG decreases, and 42% of cirrhotic patients with ascites had optimal HVPG decreases [7]. Optimal HVPG reduction is necessary for cirrhotic patients to prevent complications of cirrhosis and to prevent early mortality. In a meta-analysis by Abraldes et al., results of a multivariate analysis showed that being a non-responder was independently associated with the risk of developing variceal rebleeding, ascites, spontaneous bacterial peritonitis, and lower survival [8]. 

Given the need for optimal HVPG reduction, other therapeutic options are in need of either replacing non-selective beta-blockers (NSBB) or being an addition to NSBB. The renin-angiotensin system presents another therapeutic target, with angiotensin II receptor blockers (ARBs) like losartan showing promise in experimental studies [9,10]. Research has investigated losartan's effects on portal pressure and clinical outcomes in cirrhotic patients, revealing mixed results regarding its impact on HVPG, which is the gold-standard measure of the severity of portal hypertension. This variation was likely due to the variations in patient demographics and study designs.

Angiotensin II receptor blocker significantly influences the pathogenesis of hepatic fibrosis, vasoconstriction within the liver sinusoids, and sodium balance in portal hypertension [11]. However, angiotensin receptor blockers can cause complications such as hypotension and renal impairment [12]. The pathogenesis of fibrosis may be reduced by losartan because it could potentially inhibit the angiotensin II receptors and offer a new treatment opportunity for liver cirrhosis.

There has been one other meta-analysis/systematic review completed on the comparison of propranolol and losartan regarding their impact on HVPG [13]. This review found that losartan is equivalent to propranolol in lowering HVPG with a mean HVPG difference of -0.59 (CI: -2.73 to -3.04). Losartan usage was also associated with a statistically significant drop in MAP (mean arterial blood pressure), with the difference of 0.53 (CI: -2.47 to -3.54). Losartan was also shown to be non-inferior to propranolol in preventing portal hypertensive bleeding [RR: 2.02 (CI: 0.38 to 10.89)]. Finally, propranolol was found to have a statistically significant heart rate decrease of 18.62 (CI: 10.10 to 27.74). Although, this study did analyze the significance of propranolol versus losartan regarding wedged hepatic venous pressure (WHVP) [13].

We believe that there is a lack of high-quality evidence regarding the influence of losartan on liver fibrosis and the complications of portal hypertension. This study aims to evaluate losartan's effect on HVPG, systemic hemodynamics, renal function, and clinical events like variceal bleeding and ascites, through a systematic review and meta-analysis of the existing literature, while also assessing the management of risks associated with its use.

Disclaimer 

It should be noted that this article was pre-printed on September 27, 2024, on the medRxiv pre-print server.

## Review

Research approach

The study followed the Preferred Reporting Items for Systematic Reviews and Meta-Analysis (PRISMA) guidelines [14].

Identification and Selection of Studies

**Figure 1:**
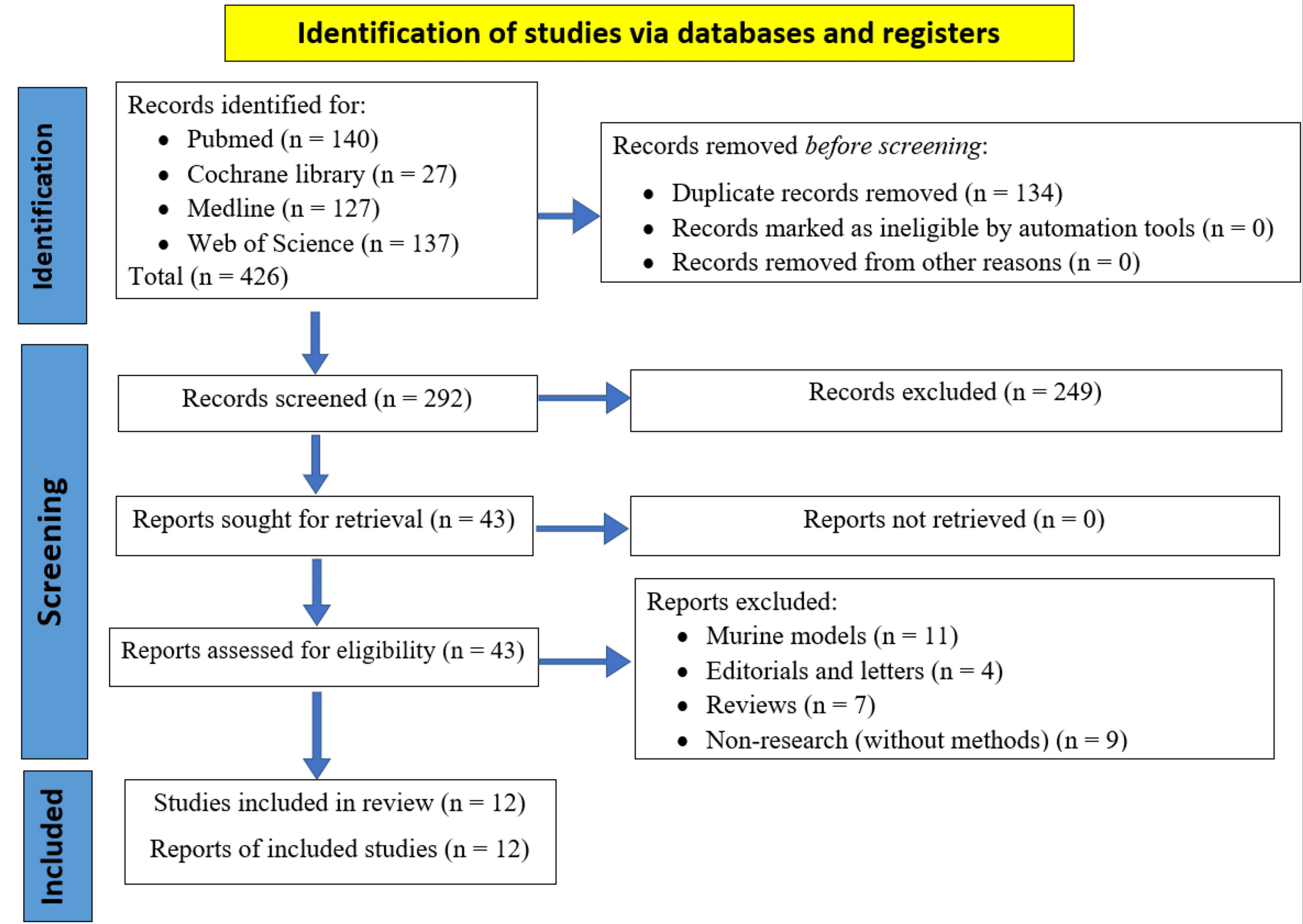
PRISMA flow diagram PRISMA, Preferred Reporting Items for Systematic Reviews and Meta-Analysis.

Literature was sourced from PubMed, Cochrane Library, Medline, and Web of Science focusing on losartan's effect on portal hypertension. Figure 1 displays the PRISMA flow diagram that shows how studies were identified, screened, and included in our meta-analysis. 

Search Strategy

Keywords used included losartan, ARB, portal hypertension, liver cirrhosis, and hepatic fibrosis (Tables 1, 2).

**Table 1:** PubMed search strings

Query	Search Details	Results
#1 AND #2	("losartan"[MeSH Terms] OR "losartan"[All Fields] OR "losartan s"[All Fields] OR "losartane"[All Fields] OR "angiotensin II receptor blocker"[All Fields] OR "ARB"[All Fields]) AND ("portal hypertension"[All Fields] OR "liver cirrhosis"[All Fields] OR "hepatic fibrosis"[All Fields])	140
("portal hypertension" OR "liver cirrhosis" OR "hepatic fibrosis")	"portal hypertension"[All Fields] OR "liver cirrhosis"[All Fields] OR "hepatic fibrosis"[All Fields]	142,986
(losartan OR "angiotensin II receptor blocker" OR ARB)	"losartan"[MeSH Terms] OR "losartan"[All Fields] OR "losartan s"[All Fields] OR "losartane"[All Fields] OR "angiotensin II receptor blocker"[All Fields] OR "ARB"[All Fields]	19,948

**Table 2:** Other database search strings

Database	Search Strings	Results
Cochrane Library	(losartan OR "angiotensin II receptor blocker" OR ARB) AND ("portal hypertension" OR "liver cirrhosis" OR "hepatic fibrosis")	27
Medline	(losartan OR "angiotensin II receptor blocker" OR ARB) AND ("portal hypertension" OR "liver cirrhosis" OR "hepatic fibrosis")	123
Web of Science	(losartan OR "angiotensin II receptor blocker" OR ARB) AND ("portal hypertension" OR "liver cirrhosis" OR "hepatic fibrosis")	137

Study Selection

Retrieved results were managed using Zotero version 6.0.36 (Corporation for Digital Scholarship, Vienna, VA), which helped exclude retracted records and merge duplicates. 

Eligibility Criteria

Research included in the study adhered to modified Population, Intervention, Comparison, Outcomes, Study Design (PICOS) criteria [15]:

Population (P): Patients with portal hypertension.

Intervention (I): Losartan.

Comparison (C): Propranolol

Primary Outcomes (O): Portal pressure, response rates, disease progression.

Study Design (S): Quantitative, qualitative, and mixed methods.

Inclusion Criteria

Peer-reviewed original research articles published in English (or translatable) on losartan's effect on portal hypertension.

Exclusion Criteria

Excluded were non-research articles, study protocols, reviews, meta-analyses, opinion pieces, conference abstracts, and editorials.

Methodological Quality Assessment: The Risk of Bias

The risk of bias was evaluated using the Robvis 2.0 tool and the Risk of Bias in Non-randomized Studies (ROBINS) [16].

The review of literature was conducted from August 20th, 2024, through August 31st, 2024. 

**Figure 2:**
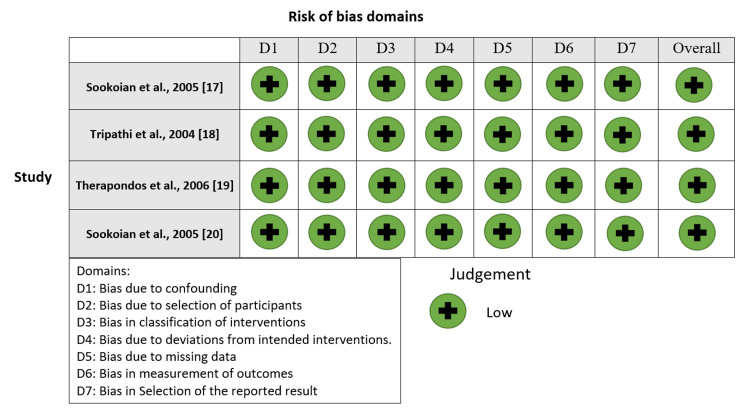
Risk-of-bias traffic light plot Traffic light plot of the Risk of Bias in Non-randomized Studies (ROBINS) with the intervention assessment results [17-20].

**Figure 3:**
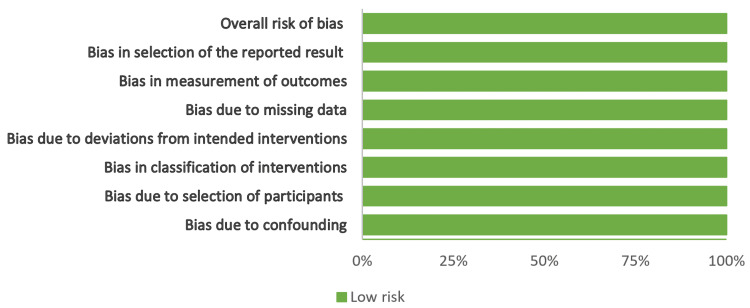
Summary plot of the risk of bias Risk of Bias in Non-randomized Studies (ROBINS) with the intervention assessment results [16-19].

**Figure 4:**
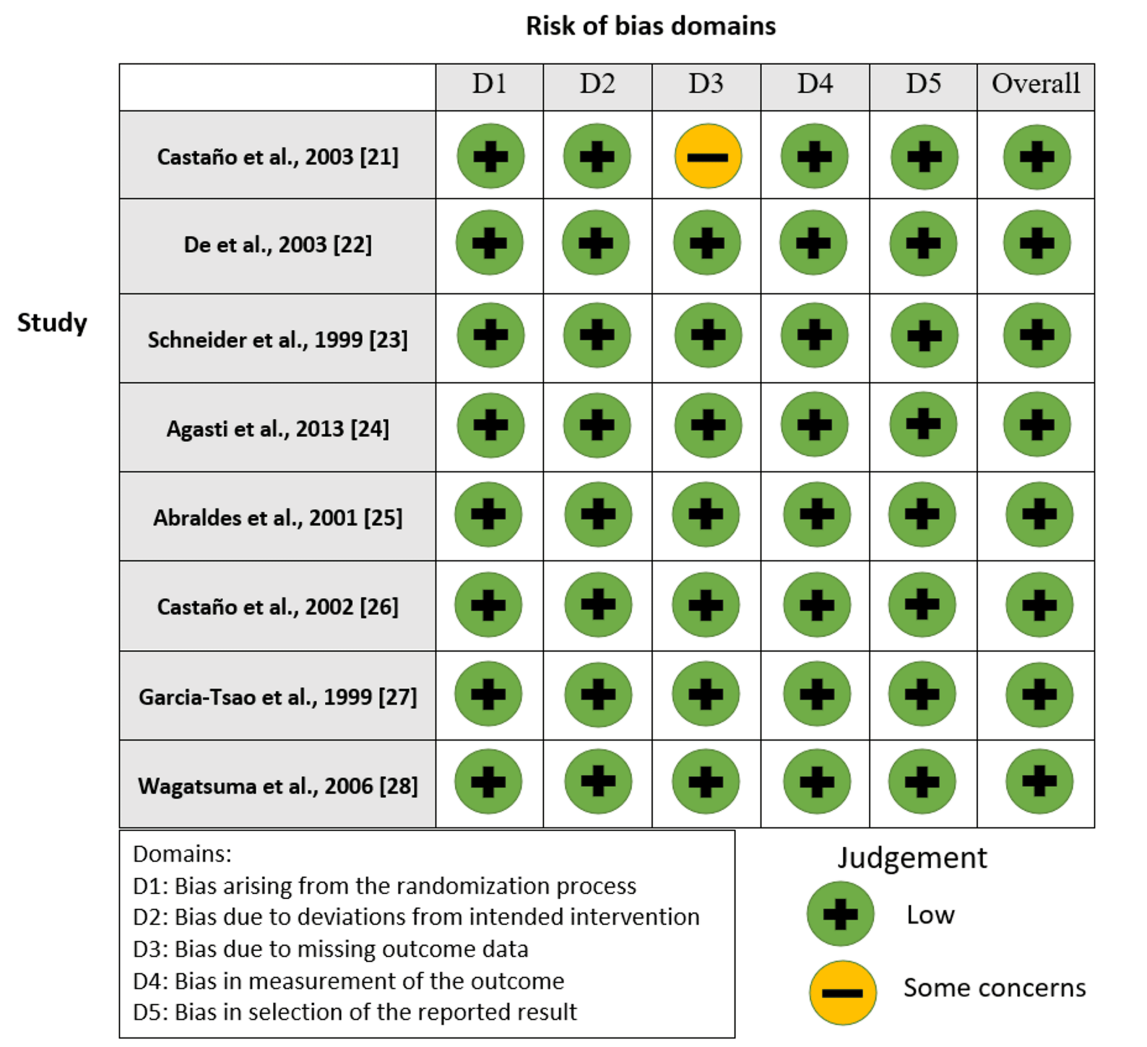
Risk-of-bias traffic light plot Traffic light plot of the risk-of-bias visualization tool assessment results [21-28].

**Figure 5:**
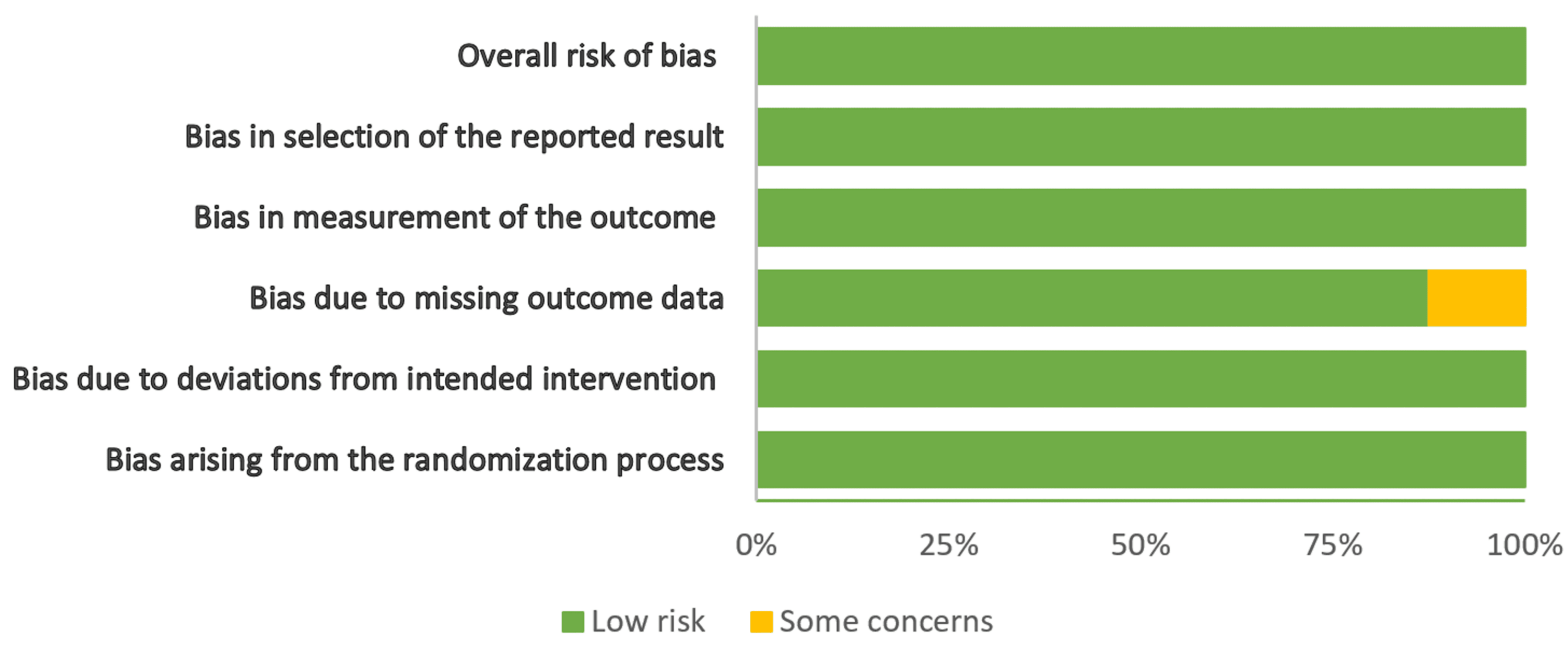
Summary plot of the risk of bias Summary plot of the risk-of-bias visualization tool assessment results [20-27].

Figures 2-5 show the risk-of-bias visualization tool (Robvis 2.0) and the ROBINS with the intervention assessment results. 

Data Extraction

Data were systematically extracted by one reviewer independently and entered manually into an Excel sheet using Microsoft Excel 2019 (Microsoft Corporation, Redmond, WA), including study ID, design, settings, sample characteristics, mean age or age range, sample size, intervention details, purpose, outcome measures, and findings. 

Data Analysis

The extracted qualitative data were analyzed and reported according to the predominant themes. On the other hand, quantitative data were analyzed using Review Manager 5.4.1 (Cochrane Collaboration, London, UK). An intervention review starting from the full review stage was used [29]. In addition, continuous data types were used, applying inverse variance statistical, random effects analysis model, and mean difference effect measure.

Study Selection

The literature search yielded 431 records, with 134 duplicates removed. Following the title and abstract screening, 255 records were excluded, leaving 42 articles for retrieval. Ultimately, 12 studies met the eligibility criteria, as illustrated in Figure 1.

Thematic analysis of outcomes and study characteristics 

The summarization of studies and analysis of outcomes can be found in Table 3. 

**Table 3:** Studies included in the analysis List of studies and their characteristics [17-28]. HVPG, hepatic venous pressure gradient; MAP, mean arterial pressure; GFR, glomerular filtration rate; WHVP, wedged hepatic venous pressure; MABP, mean arterial blood pressure; FHVP, free hepatic venous pressure; SVR, systemic vascular resistance; PTRNa, proximal tubular sodium reabsorption.

Study	Study design	Study settings	Sample characteristics	Mean age/age range	Sample size	Intervention	Comparator	Study purpose	Outcome measures	Findings
Abraldes et al., 2001 [25]	Randomized controlled trial	Spain	Patients with cirrhosis who had bled from esophageal varices	18-75 years	40	Losartan (6.25 mg-50 mg)/day	Propranolol (20 mg-160 mg) twice daily	To compare the hemodynamic and renal effects of losartan vs. propranolol in portal hypertensive patients with cirrhosis treated after a variceal bleeding episode	HVPG, systemic hemodynamics, renal function, and vasoactive factors	Losartan did not significantly reduce HVPG but decreased MAP and GFR in Child B patients. Propranolol reduced HVPG and cardiac output but did not modify MAP or renal function.
Agasti et al., (2013) [24]	Randomized controlled trial	India	Patients with Child-Pugh B cirrhosis and large varices	30-60 years	30	Losartan (12.5 mg once daily). After three days, 25 mg daily	Propranolol (40 mg daily). 20 mg increase after every three days	To compare the efficacy of losartan with propranolol on portal hypertension in patients with decompensated chronic liver disease	HVPG, WHVP, MABP, FHVP	Losartan and propranolol were equally effective in reducing portal pressure. Both groups had 40% responders. WHVP and HVPG reduction was greater in the losartan group, but no significant difference was observed between the two groups.
Castaño et al. (2003) [21]	Randomized controlled trial	Argentina	Patients with cirrhosis and endoscopically proven esophageal varices and permeability of portal vein.	18-75 years	27	Losartan (25 mg daily)	Propranolol	To compare the effectiveness of losartan versus propranolol in the treatment of portal hypertension.	Heart rate, cardiac output, and hepatic portal venous gradient.	Administration of losartan is effective in lowering portal pressure in patients with compensated cirrhosis, especially those with severe portal hypertension.
De et al. (2003) [22]	Randomized controlled trial	India	Individuals with cirrhosis and esophageal varices	Age range: 15-65 years	39	Losartan (25 mg once daily)	Propranolol (40 mg twice a day)	To evaluate the effect of losartan on portal pressure after 14 days and compare it with propranolol	HVPG reduction	Losartan showed higher response rates (78.94%) than propranolol (45%). Losartan was more effective in non-ascitic and alcohol-abusing cirrhotic patients.
García–Tsao (1999) [27]	Prospective	Germany	Patients with cirrhosis	Not specified	70	Losartan (25 mg daily oral)	No treatment	To evaluate the effect of losartan on portal pressure (HVPG)	HVPG, blood pressure, heart rate, body weight, liver and kidney function	Losartan significantly decreased HVPG in both severe (46.8%) and moderate (44.1%) portal hypertension. No significant changes in controls. Slight decrease in MABP.
Castaño et al. (2002) [25]	Randomized trial	Argentina	Cirrhotic patients with portal hypertension	Not provided	18	Losartan (25 mg/day for 12 weeks)	Propranolol	To compare the effects of losartan vs. propranolol on HVPG in cirrhotic patients with portal hypertension	HVPG, MAP, cardiac output, portal blood flow, SVR, heart rate	Losartan led to a decrease in HVPG and SVR. There was no significant change in MAP. Propranolol showed a significant decrease in HVPG.
Schneider et al. (1999) [23]	Randomized controlled trial	Germany	Cirrhotic patients with portal hypertension	Not specified	30	Losartan (25 mg daily for 1 week)	No treatment	To evaluate the effect of losartan on portal pressure in cirrhosis	HVPG, blood pressure, heart rate, body weight, liver and kidney function	Significant decrease in HVPG in losartan-treated patients with severe and moderate portal hypertension. No significant change in controls. There is a slight but significant decrease in mean arterial blood pressure.
Sookoian et al. (2005) [17]	Non-randomized longitudinal study	Argentina	Patients with cirrhosis and portal hypertension	18-75 years	23	Losartan, a dose of 25 mg daily	None	To investigate the relationship between genetic polymorphisms of the renin-angiotensin system and the effects of losartan on portal and systemic hemodynamics in patients with cirrhosis and portal hypertension.	Hepatic portal venous gradient, wedged hepatic venous pressure, free hepatic venous pressure.	A significant relationship exists between AT1R A1166C polymorphisms and the therapeutic response to losartan.
Sookoian et al. (2005) [20]	Non-randomized controlled design	Argentina	14 patients with chronic hepatitis C non-responders	49.6 ± 13 years	14	Losartan (50 mg/d for 6 months)	A control group of untreated patients	To evaluate the safety and efficacy of losartan on hepatic fibrosis in chronic hepatitis C patients	Changes in fibrosis stage and blood pressure	Significant decrease in fibrosis stage in losartan group (decrease of 0.5 ± 1.3) compared to controls (increase of 0.89 ± 1.27; p < 0.03)
Therapondos et al. (2006) [19]	Non-randomized experimental study	Canada	10 post-TIPS ascites-free patients with cirrhosis (9 male patients, 1 female patient)	52.2 ± 3.2 years	10	Single oral low-dose losartan (7.5 mg)	None	To investigate the role of posture in sodium retention in post-TIPS ascites-free patients and to study the effect of losartan on sodium handling.	Sodium excretion, PTRNa, plasma renin, angiotensin II, aldosterone levels	Losartan blunted PTRNa (supine 69.7% to 63.9%, p < 0.01; erect 81.1% to 73.8%, p < 0.01), but sodium retention remained.
Tripathi et al. (2004) [18]	Non-randomized controlled design	United Kingdom	12 patients with parasitic cirrhosis	53.8 ± 3.3 years	12	Losartan (daily administration of 25 mg)	None	To investigate the systemic and portal hemodynamics and tolerability after chronic administration of losartan.	Hepatic portal venous gradient, WHVP, MAP.	Chronic administration of low-dose losartan does not lead to a significant reduction in the portal pressure gradient.
Wagatsuma et al. (2006) [28]	Randomized experimental study	Japan	16 patients with portal hypertensive gastropathy	Not specified	16	Losartan (daily administration of 25 mg or 50 mg dose)	50 mg losartan compared to 25 mg.	To evaluate the efficacy of losartan in the treatment of portal hypertensive gastropathy.	Mean portal vein blood flow and congestion index.	Losartan was found to be effective in the treatment of portal hypertensive gastropathy.

Changes in HVPG and WHVP

Research indicated varying effects of losartan on HVPG, WHVP, and mean arterial pressure (MAP). The study conducted by Tripathi et al. showed no significant change in HVPG after four weeks of losartan treatment, with a reduction from 15.4 ± 1.5 to 13.6 ± 1.6 mmHg (p = 0.1). There was a significant reduction in WHVP, falling from 20.3 ± 1.8 to 17.3 ± 1.8 mmHg (p < 0.05) and a significant decrease in MAP, from 97 ± 3.0 mmHg to 89 ± 4.0 mmHg (p = 0.02) [18]. Another study found a significant reduction in HVPG in patients with severe portal hypertension, dropping from 24.8 ± 3.6 to 13.1 ± 4.1 mmHg (p < 0.001), compared to the control group with a minor decline from 23.9 ± 4.1 to 23.1 ± 4.2 mmHg and WHVP reduction from 22.1 ± 2.6 to 14.1 ± 2.9 mmHg (p < 0.001) in patients with moderate hypertension. The control group demonstrated a smaller reduction from 22.0 ± 2.2 to 21.4 ± 2.6 mmHg [23]. 

In other studies, losartan reduced HVPG in moderate cases by 46.8%, from 17.9 ± 1.4 to 10.0 ± 2.7 mmHg [26]. Comparatively, losartan reduced HVPG from 15.6 ± 4.2 mmHg to 11.8 ± 3.5 mmHg, while propranolol reduced it from 16.4 ± 4.1 to 13.1 ± 3.6 mmHg, showing a more significant reduction of -10% ± 11% (p = 0.003) compared to losartan's non-significant change of -2% ± 12% [21]. The study conducted by De et al. showed that losartan decreased HVPG from 19.21 ± 3.82 to 14.15 ± 4.91 mmHg, while propranolol reduced it from 18.7 ± 3.77 to 15.45 ± 5.35 mmHg. Losartan also reduced WHVP from 32.42 ± 6.61 to 28.31 ± 5.09 mmHg, and propranolol lowered it from 34.55 ± 5.41 to 32.75 ± 8.13 mmHg [22].

Losartan significantly decreased MAP from 97 ± 3.0 to 89 ± 4.0 mmHg (p = 0.02) and from 90.9 ± 5.5 to 87.4 ± 4.6 mmHg in two other studies who compared its effect with propranolol. A reduction in systolic arterial pressure from 134 ± 22.7 to 124 ± 18.1 mmHg was noted, along with minor increases in diastolic pressure and MAP. In comparison, propranolol led to significantly lower HVPG and MAP, with HVPG remaining higher in the losartan group. Losartan reduced the heart rate from 79.6 ± 1.5 to 78.1 ± 2.5 BPM, while propranolol led to a larger decrease from 80.8 ± 6.2 to 68.3 ± 5.5 BPM [17,24].

*Patient Demographics and Genetic Factors* 

The study conducted by Sookoian et al., on genetic polymorphisms of the angiotensin II type 1 receptor gene, provided some insights on the variable effect of losartan in different genotypes. According to this study, losartan significantly decreased HVPG in portal hypertension patients with genotype AA from 15.7 ± 4.3 to 10.6 ± 3.4, in contrast to patients with genotypes AC and CC, who showed minor reductions from 15.9 ± 1.6 to 15.4 ± 2.8. A similar trend was observed in WHVP, where genotype AA patients showed significant improvement from 26.0 ± 4.4 to 21.9 ± 6.1 mmHg, while no significant changes were noted in patients with AC and CC genotypes [17].

Impact on Renal Markers and Sodium Handling 

The renal function markers like glomerular filtration rate (GFR), blood urea nitrogen, and creatinine were not affected by propranolol. However, losartan significantly decreased GFR in Child B patients, particularly those with reduced MAP and systemic vascular resistance. Additionally, there were no significant changes in GFR during diuretic use [25]. The effect of losartan on sodium handling in kidneys was also noted by Tripathi et al. during their study on HVPG (quoted above). According to their findings, there was no significant change in creatinine clearance after four weeks of losartan, and it was recorded that losartan caused reduction of sodium excretion from 154 ± 61 mmol/day to 122 ± 36 mmol/day [18]. Similar findings were recorded by another study in which losartan administration resulted in decreased proximal tubular reabsorption of sodium in both supine and erect positions, with significant decreases noted in both positions (supine: from 69.7 ± 3.1% to 63.9 ± 3.9 %, p = 0.01; erect: from 81.1 ± 1.8% to 73.8 ± 2.4%, p = 0.01) [19]. 

Antifibrotic Effects

Losartan led to a decrease in the fibrosis stage, with the treated group showing a reduction of 0.64 ± 1.3 compared to an increase of 0.89 ± 1.27 in the control group. The reduction in fibrosis was further evidenced by improvements in seven out of 14 treated patients, compared to only one out of nine in the control group. Sub-endothelial fibrosis in lobular areas significantly decreased from a baseline of 2.48 ± 1.04 to 1.00 ± 0.53 after losartan treatment, while no significant changes were observed in the control group [20].

Management of Portal Hypertension-Related Complications

According to a study conducted by Wagatsuma et al., portal hypertensive gastropathy improved in nine out of 16 patients, with an efficacy rate of 56% after losartan administration. Higher efficacy was observed with a 50 mg dose of losartan (83%) compared to a 25 mg dose (40%) [28].

Safety and Adverse Effects

Losartan and propranolol were well-tolerated by over 90% of patients, though some studies reported adverse events. Transient hypotension was commonly seen after the first dose of losartan, with no recurrence during continued treatment. Mild orthostatic hypotension, gastrointestinal bleeding, and encephalopathy were also noted, with some patients developing these conditions after using either losartan or propranolol. Severe side effects included rebleeding associated with losartan, though none of the adverse events were fatal, and treatment withdrawal was necessary in only one case. In patients with severe portal hypertension, losartan also caused nausea and dizziness. The findings from the study from Abraldes JG et al. show that the overall incidence of adverse events was similar for losartan (28%) and propranolol (27%) [25].

Meta-Analysis Findings

**Figure 6:**
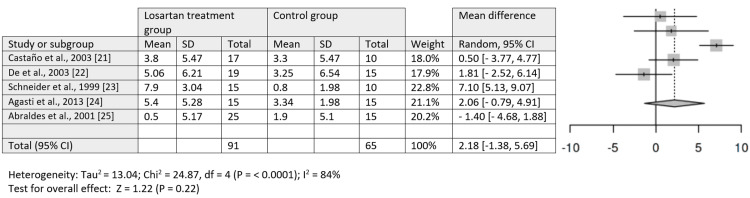
Forest plot of hepatic venous pressure gradient comparison Hepatic venous pressure gradient comparison between the losartan treatment group and the control group [21-25].

**Figure 7:**
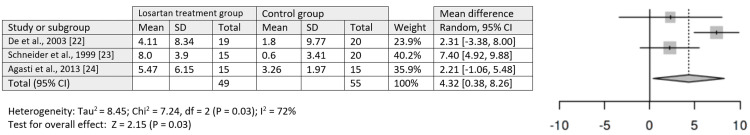
Forest plot of WHVP comparison WHVP comparison between the losartan treatment group and the control group [22-24]. WHVP, wedged hepatic venous pressure.

The meta-analysis included five studies (study numbers [20-24]) that reported quantitative data on HVPG changes. The analysis indicated no statistically significant difference in HVPG change between the losartan treatment group and the control group (p = 0.22). High heterogeneity was noted among the included studies, with an I² of 84%, likely due to variations in sample sizes and study designs. Figures 6, 7 illustrate forest plot data of HVPG comparisons and WHVP for losartan treatment groups and control groups. 

Overall, this structured analysis underscores losartan's potential efficacy in managing portal hypertension, with specific emphasis on its effects on HVPG, WHVP, and MAP, as well as its comparative efficacy against propranolol. The findings also highlight the importance of patient demographics, particularly genetic factors, and renal function in treatment outcomes. 

**Figure 8:**
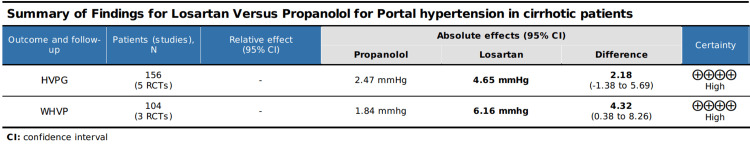
Certainty of evidence for meta-analysis HVPG, hepatic venous pressure gradient; WHVP, wedged hepatic venous pressure; RCT, randomized controlled trial.

Figure 8 illustrates the certainty of evidence using the GRADE method [30].

Discussion

This study assessed the effects of losartan on portal hypertension. The findings of this systematic review and meta-analysis indicate that managing the condition is challenging. The variable results in terms of the effectiveness of losartan for lowering HVPG across studies relate directly to the variability in the severity and baseline characteristics of portal hypertension. Some studies showed marked reductions in HVPG, while others showed negligible changes, resulting in no statistically significant overall change in HVPG in the meta-analysis. These differences could be attributed to factors such as the variation in liver disease type and extent, initial portal hypertension severity, and differences in treatment duration and dosages [31].

Further investigation into losartan's hemodynamic effects revealed changes in WHVP. However, the impact was not statistically significant, highlighting that portal hypertension's pathophysiology is multifactorial, with various mechanisms influencing the initial improvements. Most studies noted decreases in the MAP, a potential concern for cirrhotic patients with fragile hemodynamic conditions. Thus, losartan therapy must be carefully adjusted to avoid systemic hypotension.

Losartan also showed therapeutic potential in mitigating liver disease-related fibrosis, aligning with preclinical findings that suggest that losartan could inhibit hepatic stellate cell activation [32]. This variability in effect corresponds with previous studies on the renin-angiotensin system in portal hypertension, linking it to complex pathways, high intrahepatic pressure, and splanchnic vasodilation [33].

Genetic factors, such as the AT1R A1166C polymorphism, may influence losartan effectiveness, suggesting personalized treatment options for portal hypertension [34]. The significant plasticity of hepatic circulation leads to compensatory reactions that might minimize losartan's initial hemodynamic effects [35], which does not guarantee long-term outcomes.

Dose optimization remains inconclusive, as higher doses reduce portal pressure but increase the risk of systemic hypotension. The timing of intervention is also crucial, with early intervention potentially offering benefits before disease progression.

The safety profile of losartan showed good tolerability, though episodes of hypotension, reduced renal function, and gastrointestinal hemorrhage were reported. These findings correlate with the renin-angiotensin system inhibition effects in cirrhotic patients emphasizing careful patient evaluation and monitoring [36,37].

Overall, the variability in response underscores the need to consider multiple factors in managing portal hypertension, including patient characteristics, disease etiology and severity, and genetic predispositions. Losartan's antifibrotic effects point to potential therapeutic approaches in the disease's early stages. Combination therapy targeting hemodynamic and fibrotic features might enhance treatment outcomes. However, safety concerns necessitate meticulous patient selection, especially for those with unstable hemodynamics. The findings of our study build upon and confirm the broader findings of Tandon et al. regarding the effects of angiotensin-converting enzyme (ACE) inhibitors and ARBs on portal hypertension in liver cirrhosis [38]. 

Further studies should explore the factors affecting losartan response, optimal dosing, and intervention timing in liver disorders. Future research should include large randomized controlled trials (RCTs) with comparisons of losartan, propranolol, and combination of both losartan and propranolol treatments. 

Limitations of the study

The heterogeneity among included studies regarding patient populations and dosing impacts the generalizability of the findings. In addition, the variability in disease etiology, severity, and baseline portal pressures across studies limit direct comparisons through meta-analyses. Further research is needed to ensure accurate interpretation of data to guide clinical decision making.

Moreover, the lack of data on specific subgroups, such as patients with different stages of liver disease or various comorbidities, limits the generalizability of the findings of losartan's efficacy to all subgroups.

## Conclusions

This study highlights losartan's potential efficacy in treating portal hypertension. Its antifibrotic properties and ability to reduce portal pressure highlight its potential to address both hemodynamic and structural aspects of the disease, especially in the early stage of liver cirrhosis.

Losartan’s effects on MAP and natriuresis could also be relevant in managing associated cardiomyopathies in cirrhotic patients. Further research is needed to fully explore losartan's comprehensive therapeutic benefits in this complex patient population, particularly focusing on its dual role in liver and cardiovascular health.
